# Genome Sequencing of Multiple Primary Lung Cancers Harbouring Mixed Histology and Spontaneously Regressing Small-Cell Lung Cancer

**DOI:** 10.3390/jpm14030257

**Published:** 2024-02-28

**Authors:** Valentina Thomas, Ahmed Rashed, Clare Faul, Siobhan Nicholson, Vincent Young, John Hanson, Bryan T. Hennessy, Sinead Toomey, Simon J. Furney

**Affiliations:** 1 Genomic Oncology Research Group, Department of Physiology and Medical Physics, RCSI University of Medicine and Health Sciences, D02 YN77 Dublin, Ireland; valentina.thomas1@hotmail.com; 2 Centre for Systems Medicine, RCSI University of Medicine and Health Sciences, D02 YN77 Dublin, Ireland; 3 Our Lady of Lourdes Hospital, A92 VW28 Drogheda, Ireland; ahmednabil616@hotmail.com (A.R.); john.hanson@hse.ie (J.H.); bryanhennessy74@gmail.com (B.T.H.); 4 Medical Oncology Group, Department of Molecular Medicine, RCSI University of Medicine and Health Sciences, D02 YN77 Dublin, Ireland; 5 St Luke’s Radiation Oncology Network, D08 E797 Dublin, Ireland; clare.faul@slh.ie; 6 Department of Histopathology, St James’s Hospital, D08 E797 Dublin, Ireland; snicholson@stjames.ie; 7 Department of Cardiothoracic Surgery, St James’s Hospital, D08 E797 Dublin, Ireland; vyoung@stjames.ie

**Keywords:** synchronous cancers, whole genome sequencing, genomics, small-cell lung cancer, non-small-cell lung cancer, spontaneous regression

## Abstract

Up to 15% of lung cancer patients present two or more anatomically separate primary lung lesions, known as multiple primary lung cancers (MPLCs). While surgical resection or stereotactic body radiation therapy (SBRT) is the standard of care for most early-stage lung cancer cases, this may not be an option for patients with widespread tumours, highlighting the need for the improved targeted management of MPLC patients, which remains challenging. Moreover, the spontaneous regression (SR) of small-cell lung cancer (SCLC) is rare, with only four cases accounted for between 1988 and 2018. We report a rare MPLC case harbouring the mixed histology of non-small-cell lung cancer adenocarcinoma (NSCLCa) and SCLC and the SR of SCLC without treatment. The patient was diagnosed in 2015 with MPLCs, identified as NSCLCa and SCLC. In 2016, a restaging PET/CT scan prior to the start of treatment showed SCLC SR. In 2018, a further tumour was detected in the patient’s mandible, and a re-biopsy of the SCLC revealed histology consistent with NSCLCa. Whole-genome sequencing (WGS) analysis identified a high expression of programmed death ligand-1 (PDL-1) in the NSCLCa, which was treated with pembrolizumab. WGS revealed distinct genomic profiles and mutational mechanisms in MPLCs, suggesting the need for distinct targeted therapies to improve the management of MPLC patients and highlighting the importance of precision evaluation.

## 1. Introduction

Lung cancer is the leading cause of cancer death worldwide [1,2]. About 15% of lung lesions are small-cell lung cancers (SCLCs), while the remaining majority are non-small-cell lung cancers (NSCLCs), predominantly of the adenocarcinoma subtype (NSCLCa) [3,4]. These distinct clinical and histological subtypes display high molecular heterogeneity, which translates into different treatment responses: while chemotherapy shows beneficial results in the treatment of SCLCs, the standard of care for NSCLCs largely depends on the stage of the lesion, with early-stage tumours generally removed surgically or treated with SBRT, and more advanced ones treated with chemoradiation therapy [5,6]. Moreover, considerable survival benefits are provided by the administration of more specific and better-tolerated treatments in patients that exhibit specific, clinically relevant cancer biomarkers as targets for therapy [4]. Currently, several predictive biomarkers can be tested, such as the high expression of programmed death ligand-1 (PD-L1) and tumour mutational burden (TMB) for immune checkpoint inhibitors (i.e., pembrolizumab) [7,8,9,10], and *EGFR* mutations, with *ALK* and *ROS1* gene fusions for tyrosine kinase inhibitors (i.e., gefitinib, erlotinib, crizotinib) [11,12,13,14,15]. Targeted therapies are especially relevant in the context of reduced tolerance to the standard of care in cases where the patient presents with widespread lesions, such as multiple primary lung cancers (MPLCs) and/or co-morbidities, and cannot endure surgery and chemoradiation therapy [16,17]. MPLCs are diagnosed in up to 15% of lung cancer patients [17]. MPLCs are physically distinct entities that occur in the same or different lung lobes. The development of MPLCs is an interesting phenomenon because it arises from an identical germline genetic background and environmental exposure and yet results in highly heterogeneous lesions. Previous studies on the molecular and genomic data of MPLCs have found that tumours developing in this context show clonally distinct molecular profiles, distinct oncogenic alterations, and can be of a different pathological type [18,19,20,21], suggesting variable responses of MPLCs to targeted therapies, and strong implications for the therapeutic management of MPLC patients. We describe a rare case of MPLCs, harbouring a mixed histology of SCLC and NSCLCa, which we analyse through whole genome sequencing. Moreover, the SCLC showed spontaneous regression (SR), making this case study a rare opportunity to gain insight into both MPLC development and the occurrence of SR.

## 2. Case Description

A 69-year-old female patient, a smoker known to have chronic obstructive pulmonary disease (COPD) and compensated alcoholic liver cirrhosis, presented in 2015 with the exacerbation of shortness of breath (SOB). A computed tomography scan (CT scan) and positron emission tomography/CT scan (PET/CT scan) revealed bilateral lung masses, one in the right lower lobe and one in the left upper lobe, with no suspicious lymph nodes or any other lesions. Endobronchial ultrasound (EBUS) biopsies were performed in September 2015 and identified an NSCLCa (sample NSC-1) in the right lung lesion and a SCLC (sample SC) in the left lung lesion. It was decided to surgically remove the NSCLCa first, followed by surgery on the SCLC. In October 2015, right lower lobe segmentectomy and mediastinal lymph node dissection were performed. Histopathology analysis confirmed the complete resection of pT1aN0 NSCLCa. However, the patient’s slow recovery made her unfit for further surgery. In April 2016, she was referred to the medical oncology service for the consideration of chemotherapy. In May 2016, a restaging PET/CT scan prior to starting treatment showed right lung post-surgical changes and partial left lung resolution. The left apical SCLC reduced in size from 28 mm to 8 mm, and its standardised uptake value (SUV) reduced from 16.5 to 2.1. A definite improvement and SR were, therefore, observed in the absence of any local or systemic treatment. Following a discussion at the lung cancer multidisciplinary meeting, it was decided to proceed with no treatment and start PET/CT scan surveillance. A stable disease, with no evidence of progression, was observed through three PET/CT scans from August 2016 to July 2017. In February 2018, the scan revealed a left apical mass increase to 22 mm and an SUV increase to 13.3, in addition to a 1 cm lesion in the right mandible. A biopsy from the mandibular lesion showed metastatic NSCLCa (sample NSC-3) with programmed death ligand-1 (PDL-1) = 60%. A re-biopsy of her left apical lung lesion showed NSCLCa (sample NSC-2), which prompted the start of patient treatment with pembrolizumab in late 2018. A restaging CT scan in January 2019 showed stable disease based on imRECIST. Histopathological analysis indicated that tumour NSC-1 was 16 mm with moderately differentiated invasive adenocarcinoma with a 40% acinar component, 30% lepidic, and 30% solid component. It was TTF-1-positive, CK 7-positive, and CK 20-negative. SC was 28 mm clinically on the PET/CT scan; the biopsy showed small-cell lung cancer with nuclear moulding and hyperchromasia with a high nuclear-to-cytoplasmic ratio. It was positive for TTF-1, AE/3, and synaptophysin with an MIB-1 index of 90%. For NSC-2, EBUS cellular aspirate showed cohesive clusters of malignant epithelial cells consistent with NSCLC. It was EGFR-negative, ALK-negative, and ROS-1-negative. NSC-3 was consistent with NSCLC with positive TTF-1, CK7, and Ber EP4. It was negative for Chromogranin, synaptophysin, CK5, 6, and CD56.

With the patient’s consent, it was decided to analyse the whole genome sequences (WGSs) of her four tumour samples (see Supplementary Methods). We identified the somatic single nucleotide variants (SNVs), small insertions and deletions (InDels), copy number alterations (CNAs), and structural variants (SVs) in the four tumour genomes to understand the different classifications of the left apical lung lesion (SCLC and NSCLCa), and to establish whether any genomic evidence existed for a potential transformation from SCLC to NSCLCa. Also, the results held potential benefits for the patient’s treatment if targetable driver mutations were identified.

**Figure 1:**
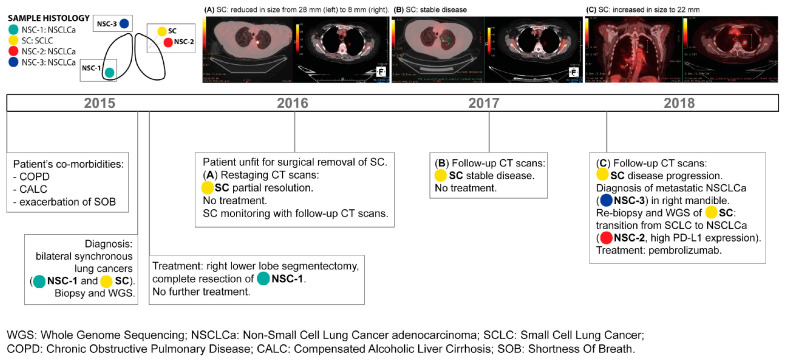
Timeline illustrating the clinical history of the patient diagnosed with multiple primary lung cancers and a potentially metastatic lesion in the mandible.

Sadly, the patient subsequently developed autoimmune acute renal failure and passed away in 2019. A timeline of the patient’s clinical history is shown in Figure 1, and clinicopathologic data of the patient’s tumours are provided in Table 1.

### 2.1. Mutational Load, Mutational Overlap and Driver Analysis of SNVs, InDels and SVs

**Figure 2:**
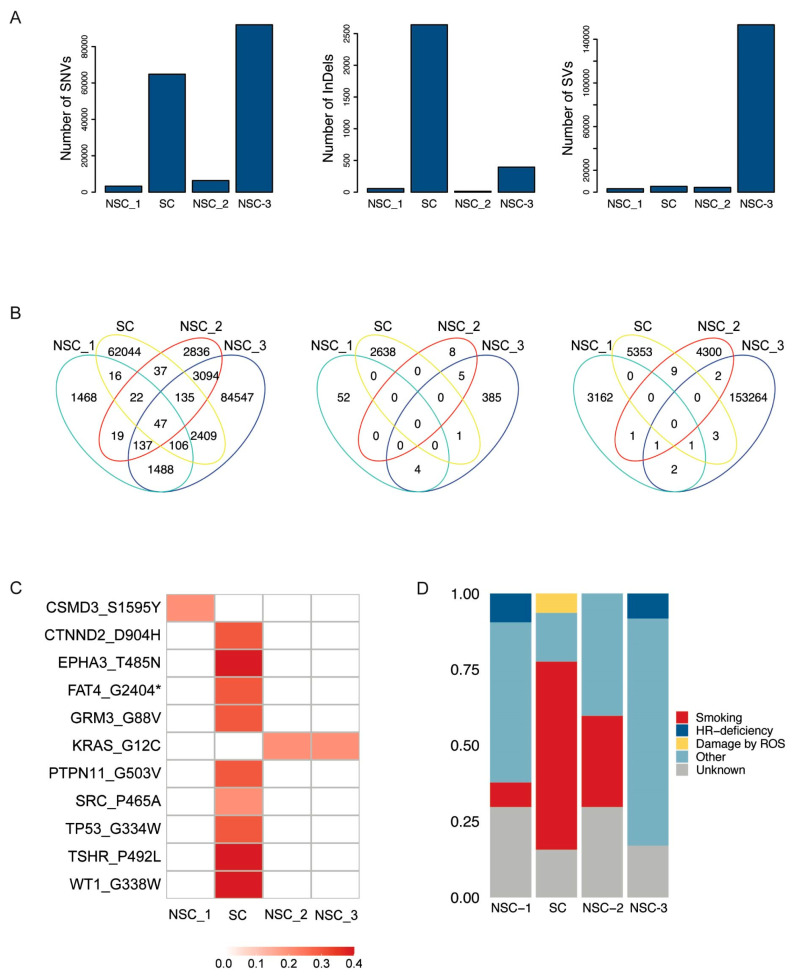
Mutational burdens, mutational overlap, putative drivers, and mutational signatures of the tumours diagnosed in the patient. (**A**) Mutational burdens of SNVs, InDels, and SVs quantified by WGS; (**B**) Venn diagrams of SNVs (left), InDels (centre), and SVs (right) show the overlap of mutations between samples; (**C**) VAFs of putative driver mutations and clinically relevant alterations; (**D**) tumour mutational profiles of total SNVs, defined by the weighted contributions of each input reference signature from COSMIC and identified by WGS. Signatures found in the samples include a “Smoking” signature (SBS4), “HR-deficiency” signature (SBS3), “Damage by Reactive Oxygen Species” signature (SBS18), and “Other” signatures (SBS12, SBS33, SBS37, SBS39, SBS40, SBS46).

The NA WGS analysis of somatic variants revealed significant differences in mutational burdens across samples and between variant types. The SNV load was higher in NSC-3 (91,963) and SC (64,816) and much lower in NSC-1 (3303) and NSC-2 (6327). The InDel burden peaked in SC (2639), followed by NSC-3 (395), NSC-1 (56), and NSC-2 (13). The SV burden was highest in NSC-3 (153,274) and dropped in SC (5368), NSC-2 (4314), and NSC-1 (3167) (Figure 2A). The vast majority of somatic mutations were unique to each sample, with only 0.03% of SNVs common to all, suggesting that the primary lesions in the patient are genetically distinct and likely to have originated independently. The highest overlap of SNVs (1.95%) and InDels (0.16%) was seen between NSC-2 and NSC-3 (Figure 2B), which included a *KRAS* G12C mutation. *TP53* was mutated in SC only (Figure 2C).

### 2.2. Mutational Signatures

Mutational signature analysis was performed to inform the exposures and biological history of each cancer and investigate the mutational processes that occurred during tumour development. Evidence of the smoking-related signature SBS4 was found in NSC-1, SC, and NSC-2 but not in NSC-3. NSC-1 and NSC-3 show evidence of the homologous recombination (HR)-related signature SBS3, though no mutations in HR-associated genes were found. SBS18, linked to damage by reactive oxygen species, was found in SC. The majority of the identified signatures, however, are of unknown aetiology (SBS12, SBS33, SBS37, SBS39, SBS40) or the result of possible sequencing artefacts (SBS46) (Figure 2D).

### 2.3. Copy Number Alterations and CNA Driver Analysis

**Figure 3:**
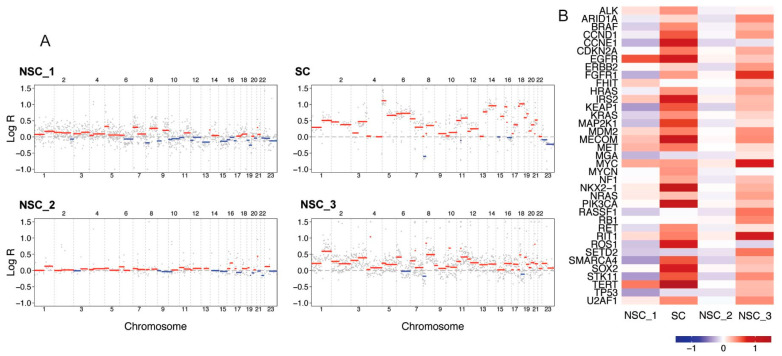
Copy number alteration analysis of the tumours diagnosed in the patient. (**A**) The genome landscapes show CNAs estimated by WGS. The *x*-axis coordinates represent positions along the genome, with vertical bars indicating the borders between chromosomes. The *y*-axis represents the chromosomal copy number log ratio in the tumour. Amplifications are depicted in red, and deletions in blue. Estimates of tumour ploidy are as follows: 2.2 for NSC-1, 3.3 for SC, 2.1 for NSC-2, and 3.2 for NSC-3. Estimates of tumour purity are as follows: 0.3 for NSC-1, NSC-2, and NSC-3, and 0.6 for SC; (**B**) Median log ratios of putative driver CNAs. Positive log ratios (gains) are represented in red, and negative log ratios (losses) are represented in blue.

Copy number alteration analysis revealed broadly stable genomes in the lung adenocarcinomas NSC-1 and NSC-2 (ploidy: 2.2 and 2.1, respectively) and higher chromosomal instability (CIN) in samples SC and NSC-3 (ploidy: 3.3 and 3.2, respectively) (Figure 3A). The copy number state of a set of known lung cancer genes is shown in Figure 3B and further highlights the heterogeneity of the samples.

## 3. Discussion

In this report, we describe an extremely rare case of SR in MPLC. This case presents the following several uncommon features: the development of histologically distinct MPLCs; a possible transition from SCLC to NSCLCa; and the SR of the SCLC.

We performed WGS on the four tumour samples diagnosed in the patient to establish the level of heterogeneity and the clonal relationship between the MPLCs and found evidence of clinically actionable alterations. We observed high genomic heterogeneity and distinct known cancer-associated events between all MPLC samples, suggesting independent origins in a carcinogen-damaged field. A *KRAS* G12C mutation, associated with response to KRAS inhibitors as well as resistance to tyrosine kinase inhibitors and poorer prognosis [22], was found in NSC-2 primary and NSC-3 mandible adenocarcinomas. This common driver might suggest that the mandibular tumour in the patient originated from the primary adenocarcinoma NSC-2; however, more evidence is needed to support this claim, especially as the *KRAS* G12C mutation is a frequent driver event that could have developed independently in each sample. *EGFR* amplification, associated with poor prognosis [8,11], was reported in all samples. *MET* amplifications were seen in NSC-1, SC, and NSC-3 and are associated with poor prognosis, though several MET inhibitors might show beneficial results in the NSCLC treatment [23,24]. The mandibular NSCLCa lesion carried the highest TMB, which, together with the high levels of PD-L1, makes it a good candidate for immunotherapy with pembrolizumab [7,10]. Overall, the scarcity of potential tumorigenic events found among known lung cancer driver genes in the patient might indeed be indicative of the occurrence of further drivers—genetic, epigenetic, or transcriptomic—that are yet to be identified.

A likely driving factor for developing bilateral synchronous lung malignancy, in particular SCLC, was the patient’s smoking history. While there is a known association between COPD and lung cancer, we were not able to directly correlate her stable COPD (or liver disease) to her primary cancer diagnosis. After primary surgery, the patient was on follow-up for her SCLC, maintained a healthy lifestyle, and had been a non-smoker for several years. We were not able to attribute the later recurrence and progression of her disease to her known medical and clinical history.

Due to the patient’s known exposure to tobacco smoke and as a result of the mutations induced by its associated carcinogens, we expected all lung samples to have high TMB. However, TMB varied greatly between the tumours, and the two lung NSCLCa samples displayed surprisingly low TMB. Evidence of the smoking-related signature (SBS4) was confirmed by mutational signature analysis in all primary lung tumours but not in the mandibular lesion. This is an interesting result because, although previous reports highlight the key role of tobacco smoke-related mutations in tumour initiation but not in tumour progression and the later stages of tumour development [25], we would still expect metastatic cancer cells to carry mutations that originate in their primary lesion [26].

While the histological transformation from adenocarcinoma to SCLC has been previously observed in the context of acquired resistance to EGFR-targeted therapies [27], the change from SCLC to NSCLCa is extremely unusual. The highly heterogeneous genomic profiles and the presence of the *TP53* mutation in SC but not NSC_2 appear to rule out the hypothesis of a phenotypic transformation from SCLC to NSCLCa. Indeed, it is possible that what we initially considered as a phenotypic transformation might have been, as observed in a small subset of lung cancers [28], a tumour harbouring the combined histology of SCLC and adenocarcinoma, the co-existence of which was not detected by the two biopsies of the lesion. Nevertheless, this finding has strong implications in clinical practice, where the potential of histologic diversity should be accounted for and where repeat biopsies might be crucial to establish the most appropriate treatment strategy.

SR, defined by Everson and Cole as the partial or complete disappearance of a malignant tumour in the absence of all treatment or in the presence of therapy which is considered inadequate to exert a significant influence on neoplastic disease, is rarely observed in malignancy [29,30]. Kumar later developed a “modified Everson and Cole criterion”, which was defined as the partial or complete disappearance of the tumour in the absence of all systemic or local treatment of the primary or metastatic lesion [31]. Based on the modified criterion, between 1951 and 2008, only five cases of primary thoracic malignancies showed SR, none of which were SCLC [31]. Between 1988 and 2018, 14 cases showed true SR with lung primary origin, only 4 of which were SCLC [29].

Our case fulfils Everson and Cole’s original and modified criteria. Our patient experienced the SR of her primary SCLC without any local or systemic treatment. This makes her one of the very few cases reported in the last 70 years [29,31]. Factors that stimulate an immune response, such as infection, trauma, surgery, and blood transfusions, have been proposed as potential explanations for SR, though the underlying mechanisms of this phenomenon remain unclear [29,31,32,33,34,35].

This study also reveals the genomic heterogeneity between the mandibular NSC-3 tumour and the primary cancers in the patient in terms of mutational overlap, driver events, and mutational processes, thus questioning its metastatic nature [26]. The analysis of multi-region biopsies should be considered in the future, as this might provide key information on the evolutionary relationship between lesions. As reported in other cancer types [36], patients with multiple primary lesions present complex scenarios that need to be carefully assessed. Our study revealed the status of several known targetable biomarkers for each MPLC, providing essential information for the personalised treatment of the patient.

## 4. Conclusions

In conclusion, we presented an in-depth analysis of a rare case of MPLCs and SR. Our study of MPLCs within a patient highlights the heterogeneity of both the genomic landscape and the lung cancer biomarkers between multiple primary tumours, which could have strong implications for therapeutic management. The occurrences of MPLCs and SR are unusual and poorly understood phenomena, warranting further investigation of their underlying mechanisms.

## Figures and Tables

**Table 1:** Clinicopathologic data of multiple primary tumours from a lung cancer patient.

Sample	Tumour Subtype	Tumour Location	Tumour Stage	Diagnosis and Biopsy	Sequencing Method	Treatment
NSC-1	NSCLCa	Right lower lobe	pT1aN0	2015	WGS	Surgical removal
SC	SCLC	Left upper lobe	cT1cN0	2015	WGS	None
NSC-2	NSCLCa	Left upper lobe	cT1cNx	2018	WGS	Pembrolizumab
NSC-3	NSCLCa	Right mandible	cT1cNxM1b	2018	WGS	Pembrolizumab

## Data Availability

The datasets supporting the conclusion of this article are available through the Genomic Oncology Research Group Data Access Committee. The names of the repository/repositories and accession number(s) can be found below: European Genome-Phenome Archive (https://ega-archive.org/—Project ID: EGAC00001001585; accessed on 10 December 2023).

## Supplementary Materials

The following supporting information can be downloaded at: https://www.mdpi.com/article/10.3390/jpm14030257/s1, Supplementary Methods.
