# Supplementary material for: Genome Sequencing of Multiple Primary Lung Cancers Harbouring Mixed Histology and Spontaneously Regressing Small-Cell Lung Cancer
Source: J Pers Med. 2024 Feb 28;14(3):257. doi: 10.3390/jpm14030257 (PMC10971334; doi:10.3390/jpm14030257)
Supplement: Supplementary file 1 [file jpm-14-00257-s001.zip › jpm-2797561-supplementary.pdf]

Supplementary Methods

Clinical sample

Four tumour samples were collected from one treatment naïve, sporadic lung cancer patient at Our Lady of Lourdes Hospital in Drogheda. Fresh tumour tissues were obtained from surgical biopsy specimens, while normal tissue was taken from the blood. Clinicopathological data describe the patient as a 69 years old female smoker with several co-morbidities, including chronic obstructive pulmonary disease (COPD), compensated alcoholic liver cirrhosis and exacerbation of shortness of breath.

DNA extraction

DNA extraction of frozen tissue was performed at Beaumont Hospital in Dublin. DNA was extracted from the tumour samples using an AllPrep DNA mini kit (Qiagen, Hilden, Germany), and from whole blood samples using a DNA blood mini kit (Qiagen), according to the manufacturers protocol. DNA was quantified by Qubit fluorometer (Invitrogen, Carlsbad, CA, USA) and DNA integrity was examined by agarose gel electrophoresis.

Whole-Genome Sequencing

A total of four tumours were diagnosed in the patient and selected for WGS, together with a matched normal sample from the patient's blood. Paired end sequencing reads (151 bp) were generated using Illumina HiSeq X sequencing technology, yielding ~30× coverage per sample. Sequences were aligned to the human reference genome (GRCh37) using BWA (Li and Durbin, 2009). PCR duplicates were marked using Picard Tools (http://broadinstitute.github.io/picard) and InDel realignment and base quality recalibration were conducted with the Genome Analysis Toolkit (GATK) v3 (http://www.broadinstitute.org/gatk).

Mutation discovery

Somatic mutations were identified by comparing each tumour sample with blood tissue as a matched normal.

Substitutions

SNVs were identified with mutation calling algorithms Strelka v1 (Saunders et al., 2012) and SomaticSniper v1.0.5.0 (Larson et al., 2012). We used BEDTools (Quinlan and Hall, 2010) to intersect their outputs, and only retained mutations found by both callers. These were further intersected with the dbSNP list of common variants (https://www.ncbi.nlm.nih.gov/SNP/) to exclude potentially germline variations. To ensure that no cancer-associated variations were removed, mutations reported in the COSMIC database (https://cancer.sanger.ac.uk/cosmic) were previously excluded from the dbSNP list. We calculated the variant allele frequency (VAF) of each SNV and further validated mutations by only keeping the ones that met the following parameters: normal alternate allele ≤ 1, minimum combined depth = 20, minimum alternate depth = 2 and minimum VAF = 0.05.

InDels

InDels were identified with Strelka v1 (Saunders et al., 2012) and filtered from potentially germline variants in the same way as the substitutions (see above).

Structural variants

SVs (deletions, tandem duplications, inversions, translocations) were identified using DELLY v0.7.9 (Rausch et al., 2012).

Copy number alterations

CNAs and tumour ploidy and purity were identified using the R package FACETS v0.5.11 (Shen and Seshan, 2016) and visualised with the R package copynumber v1.26.0 (Nilsen et al., 2012).

Gene annotation and driver analysis

The genic location and functional impact of SNVs, InDels and SVs were annotated using the Ensembl Variant Effect Predictor (VEP) v97 (McLaren et al., 2016). Known driver genes (Cancer Genome Atlas Research, 2014) together with HR DNA repair genes were searched for causative mutations in all samples. This was done through the Cancer Genome Interpreter (Tamborero et al., 2018) (https://www.cancergenomeinterpreter.org/home) and VEP (McLaren et al., 2016). The VAF of each identified driver was calculated to establish its prevalence. CNAs were annotated using the annotate_variation function implemented by ANNOVAR v2019Oct24 (Wang et al., 2010) and searched for drivers based on known NSCLCa and SCLC-associated somatic gene CNA (Cancer Genome Atlas Research, 2014). The relevance of each putative driver CNA was estimated through its median log-ratio, which was provided by the FACETS analysis (Shen and Seshan, 2016).

Mutational overlap with Venn diagrams

The overlap of SNVs, InDels and SVs between the tumours within the patient was calculated and visualised with Venn diagrams using the R package VennDiagram v1.6.20 (Chen and Boutros, 2011).

Mutational signature analysis

Mutational signature analysis was performed to inform on the exposures and biological history of a cancer. Mutational signatures were identified from SNVs using the R package deconstructSigs v1.9 (Rosenthal et al., 2016) based on the pan-cancer catalogue of single base substitution (SBS) signatures referenced in the COSMIC v3 database (https://cancer.sanger.ac.uk/cosmic/signatures).
